# Transcriptome Analysis of Wheat–*Tilletia indica* Interaction Provides Defense and Pathogenesis-Related Genes

**DOI:** 10.3390/plants11223061

**Published:** 2022-11-11

**Authors:** Malkhan Singh Gurjar, Shekhar Jain, Rashmi Aggarwal, Mahender Singh Saharan, Tej Pratap Jitendra Kumar, Lalit Kharbikar

**Affiliations:** 1 Division of Plant Pathology, ICAR-Indian Agricultural Research Institute, New Delhi 110012, India; 2 Faculty of Life Sciences, Mandsaur University, Mandsaur 458001, India; 3 Biotechnology Section, ICAR–National Institute of Biotic Stress Management, Raipur 493225, India

**Keywords:** Karnal bunt, *Tilletia indica*, wheat, DEGs, RNA Seq, defense genes, mapping

## Abstract

Karnal bunt (*Tilletia indica* Mitra) is an internationally quarantined disease of wheat. Until now, very little information has been available on the molecular basis of resistance and pathogenicity of *T. indica*. To investigate the molecular basis of host–pathogen interaction, the transcriptome of *T*. *indica* inoculated resistant (HD29) and susceptible (WH542) genotypes of wheat were analyzed. Approximately 58 million reads were generated using RNA sequencing by the Illumina NextSeq500 platform. These sequence reads were aligned to a reference genome of wheat to compare the expression level of genes in resistant and susceptible genotypes. The high-quality reads were deposited in the NCBI SRA database (SRP159223). More than 80,000 genes were expressed in both the resistant and susceptible wheat genotypes. Of these, 76,088 were commonly expressed genes, including 3184 significantly upregulated and 1778 downregulated genes. Four thousand one hundred thirteen and 5604 genes were exclusively expressed in susceptible and resistant genotypes, respectively. Based on the significance, 503 genes were upregulated and 387 genes were downregulated. Using gene ontology, the majority of coding sequences were associated with response to stimuli, stress, carbohydrate metabolism, developmental process, and catalytic activity. Highly differentially expressed genes (integral component of membrane, exonuclease activity, nucleic acid binding, DNA binding, metal ion binding) were validated in resistant and susceptible genotypes using qPCR analysis and similar expression levels were found in RNA-Seq. Apart from the wheat, the mapping of *T*. *indica* was 7.07% and 7.63% of resistant and susceptible hosts, respectively, upon infection, which revealed significant pathogenesis-related genes. This first study provided in-depth information and new insights into wheat–*T*. *indica* interaction for managing Karnal bunt disease of wheat.

## 1. Introduction

Wheat (*Triticum aestivum*, AABBDD 2n = 42) is the most widely grown crop in the world, including in India, where its production has reached to 107.59 million tons with 3421 kg/ha productivity [1] (Anonymous, 2020–21). India is the second largest producer of wheat in the world. Among biotic factors, Karnal bunt disease is the most important restriction, causing huge monetary losses in its trade caused by a hemibiotrophic fungus *Tilletia indica*. It was first reported from the Karnal district of India in the year of 1931 [2]. It has since been found in major wheat-growing states of India, as well as in other countries [3]. The pathogen is an international quarantine fungal pathogen. The disease has been reported in many countries, such as Afghanistan, Pakistan, Nepal, Mexico, some parts of the United States, Iraq, Iran, Lebanon, Syria, Sweden, Turkey, and South Africa. Presently it is a major biosecurity concern to export wheat [4,5,6]. The disease has been warned of due to climate change conditions in coming years [7].

*T. indica* is a soil, seed, and air-borne fungus, which infects mainly the floral parts of wheat [8,9]. Identification of Karnal bunt disease in the field is complex because the symptoms are not very evident. The distinctive symptom of bunt sori development is only on a few grains in the head instead of the whole head [10]. Another peculiar symptom is that infected grains emit a rotten fishy smell due to the presence of the trimethylamine compound [11,12]. Seed- or soil-borne teliospores seem to initiate in Karnal bunt infection [13,14,15].

Genetic diversity in *T*. *indica* was thought to be due to its genetic recombination [16,17,18]. Recently, a few draft genomes of *T*. *indica* has been sequenced, and putative virulence genes were validated [19,20,21]. Still, there is a need for complete genome sequencing of *T*. *indica*. The screening of wheat genotypes against Karnal bunt is very challenging. Karnal bunt-resistant wheat cultivars can reduce the severity of the disease. However, the development of resistant cultivars is very difficult since the pathogen-resistant genetic architecture in wheat is not fully identified. Understanding the molecular mechanisms of the pathogen during the infection process is essential for identifying targets for disease management [22]. Few pathogenesis-related genes (*TiHog1*, *Ti57*, and *Ti198*) have been validated in *T*. *indica* under *in planta* conditions [23].

Keeping this in view, we initiated the transcriptome analysis to understand mechanism and pathways during wheat-*T*. *indica* interactions in both resistant and susceptible wheat genotypes. The transcriptome analysis aimed to identify in three ways (i) the wheat genes, which are differentially expressed in both resistant and susceptible genotypes, respectively, (ii) the genes which are associated with the defense mechanism, and (iii) the identification of virulence related genes in *Tilletia indica*. Comparative transcriptome analysis has revealed the role of host genes, which exclusively regulate the infection in resistant as well as susceptible wheat genotypes.

## 2. Results

### 2.1. RNA Sequence Analysis and Mapping with Reference Wheat Genome

RNA-seq generated more than 5 Gb of transcriptome data from each library constructed out of a resistant and susceptible genotype, respectively. The high-quality reads were deposited in the NCBI SRA database (SRP159223). The average library fragment size was 473 bp to 504 bp. The average GC contents in the libraries were 51.38% and 49.48% for resistant (HD29) and susceptible genotypes (WH542), respectively. Resistant and susceptible genotypes generated 29,321,161 and 29,062,661 high-quality reads, respectively. About 94 percent of reads from resistant (HD29) and 92 percent of reads from susceptible genotypes (WH542) aligned to a wheat reference genome (Table 1). The remaining 6 percent of reads from the resistant genotype and 8 percent of reads from the susceptible genotype remained unmapped. 

### 2.2. Differential Gene Expression Patterns

**Figure 1:**
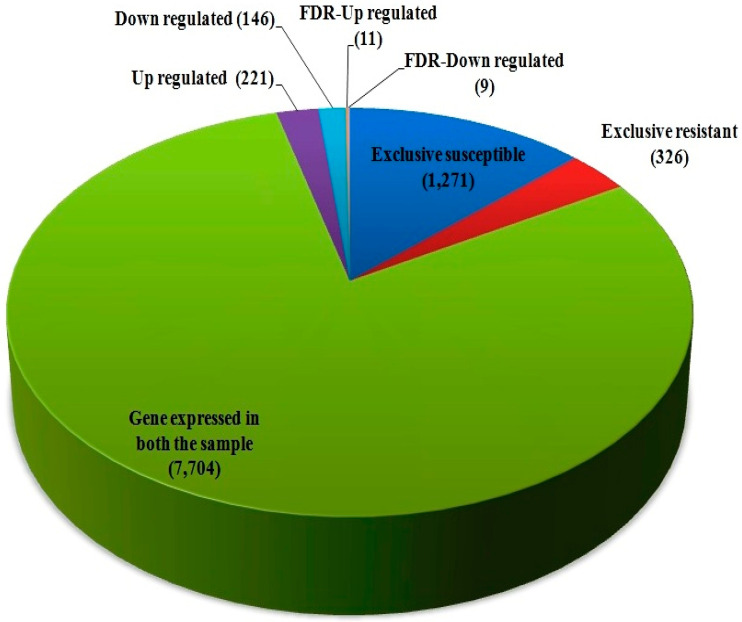
Differentially expressed genes (DEGs) between susceptible (WH542) and resistant (HD29) genotypes of wheat.

**Figure 2:**
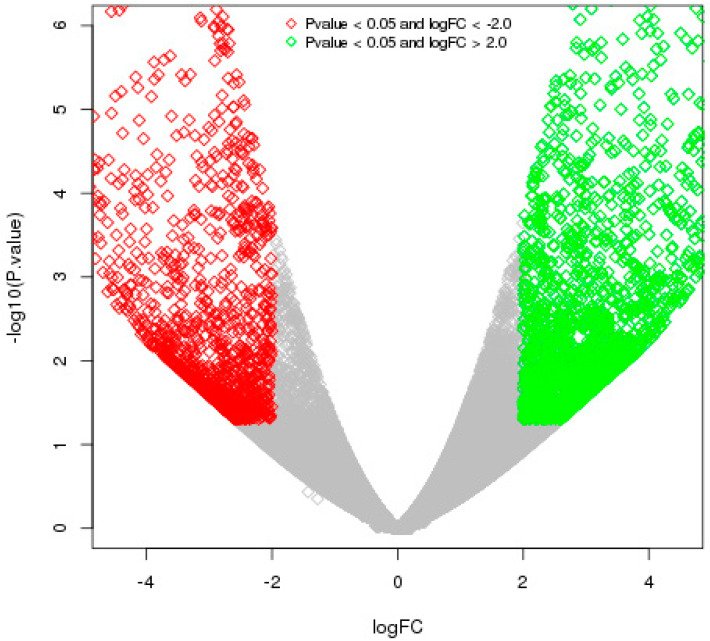
A volcano plot showing the distribution of DEGs among resistant and susceptible genotypes of wheat upon *T. indica* infection. DESeq analysis was performed to show the distribution of DEGs. Green spots correspond to genes with *p*-value < 0.05 and logFC > 2, while red spots correspond to genes with *p*-value < 0.05 and logFC <−2.

**Figure 3:**
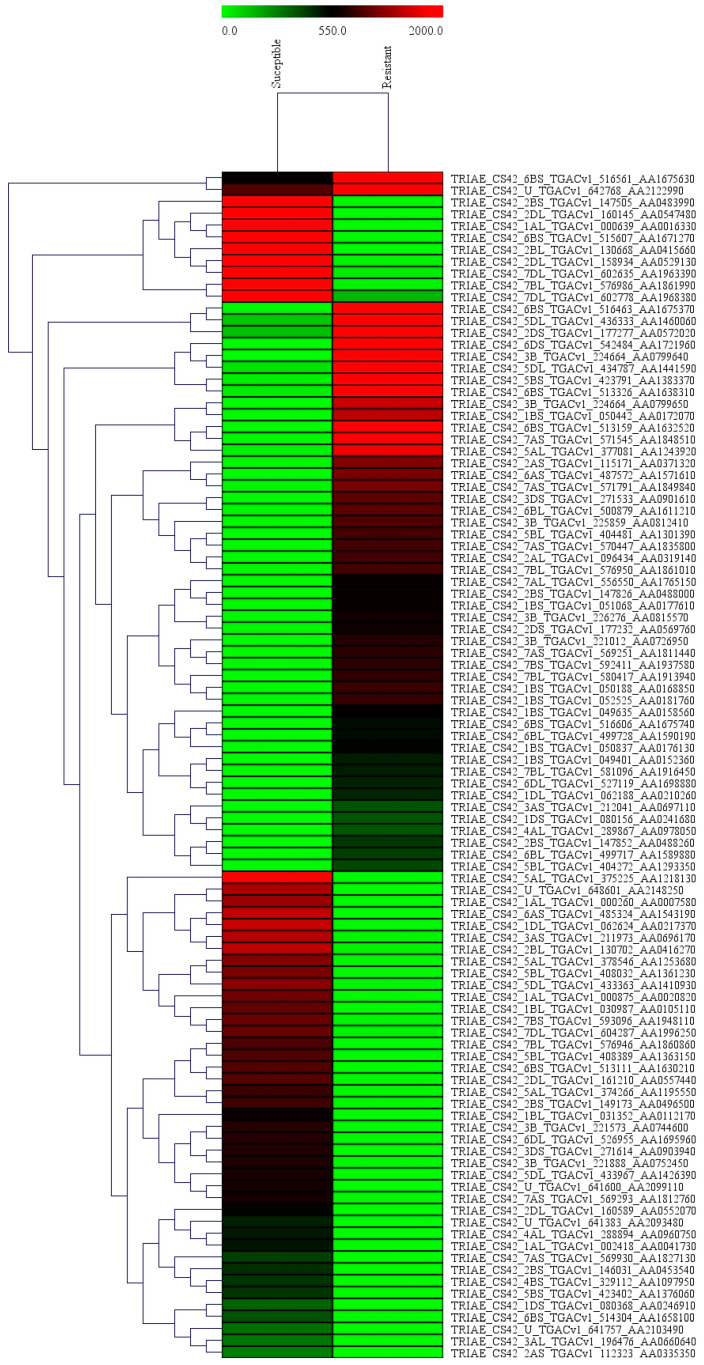
A Heat map of differentially expressed genes of *T*. *indica*. The color codes (red for upregulated genes and green for downregulated genes) are shown to indicate the relative expression of DEGs.

The differentially expressed genes were recognized through the libraries constructed from *T. indica*-infected susceptible (WH542) and resistant (HD29) wheat transcripts. In total, 76,088 genes were found to be expressed in both the susceptible and resistant genotypes. In comparison of *T. indica*-infected resistant and susceptible genotypes, 3184 (around 4.2%) genes were found to be upregulated, while 1778 (around 2.4%) genes were found to be downregulated. On the other hand, 5604 and 4113 genes were found to be exclusively expressed, respectively, in resistant (HD29) and susceptible (WH542) genotypes, while 66,371 were found to be commonly expressed (Figure 1). Further study based on the FDR showed that 503 genes were significantly upregulated and 387 significantly downregulated at a cut-off of 10% FDR (Table S1). In the volcano plots illustration, a red block showed the significantly upregulated genes, whereas a green block represented the significantly downregulated genes. A gray block represented the non-differentially expressed genes (Figure 2). Relatively high expression values for upregulated DEGs (indicated in red colour) compared to low expression values for downregulated DEGs (indicated in green colour) were obtained (Figure 3).

### 2.3. Gene Ontology Enrichment Study of DEGs and Pathways Analysis

**Figure 4:**
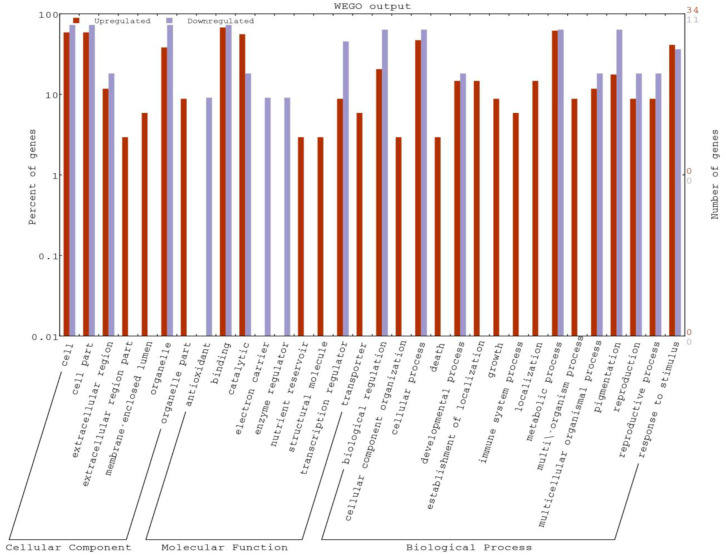
Representation of differentially expressed genes (DEGs) in a WEGO plot.

The gene ontology analysis was carried out to evaluate the potential DEG functions. The GO analysis revealed the GO term representations in both the genotypes. In the cellular component category, most of the DEGs were associated with cell, cell part, extracellular parts, and organelle. Meanwhile, in the molecular function categories, the high numbers of DEGs were associated in binding and catalytic activity as well as transcription regulators. In the third category, i.e., biological process, DEGs related to metabolic process, cellular processes, biological regulation, and response to stimulus were localized. Overall, the GO analysis indicated that most of the DEGs were involved in the biological regulation, and metabolism. Most of the coding sequences (CDS) were associated with response to stimuli (abiotic and chemical), response to stress, carbohydrate metabolism, developmental process, and catalytic activity (Table 2, Figure 4).

In total, 392 GO terms were significantly expressed in both genotypes (Table S2). Importantly, two significant GO terms, involved in responses to stimulus (GO: 0050896) and stress (GO: 0006950), were obtained for genes expressed only in resistant genotype (Table S3), while four significant GO terms involved in biological regulation (GO: 0065007), regulation of biological process (GO: 0050789), cellular biosynthetic process (GO: 0044249), and biosynthetic process (GO: 0009058) were obtained for genes expressed only in susceptible genotypes (Table S4). Out of total GO terms, 15 GO were significantly upregulated, while 40 GO were significantly downregulated. In addition, KEGG pathway mapping was also carried out, and the results showed that 208 pathways were identified under 22 functional categories (Table S5). The defense-related genes altered after the infection of the wheat with *T*. *indica*. One hundred eighteen defense-related genes were identified in the global transcriptome analysis. The expression of the gene encoding proteins was responsible for the pathogen recognition, defense response to fungus, response to biotic stimulus, metal ion binding, and defense reinforcement. The pathogenesis-related genes viz. chitinase, peroxidase, and glucanase genes altered during *T*. *indica* infection (Table S6). 

### 2.4. Mapping and DEGs in T. indica Transcripts

High-quality reads were mapped with *T. indica* RAKB_UP_1 reference genome sequence for *in silico* proof of wheat infection by *T. indica;* 7.07% reads of resistant genotype, while 7.63% reads of susceptible genotype were mapped. (Table 3). In total, 7704 commonly expressed genes of *T. indica* were obtained, which includes 221 significantly upregulated and 146 significantly downregulated genes (Table 4, Table S7). One thousand two hundred sixty-seven and 326 genes of *T. indica* were exclusively expressed in the susceptible and resistant genotypes, respectively. Based on FDR value, 11 genes were significantly upregulated viz. the oxidation–reduction process, translation, and metabolic process, and nine genes were significantly downregulated viz. response to stress, intracellular transport, and single organism biosynthetic pathway (Table 4). 

### 2.5. Validation of Differentially Expressed Genes (DEGs) Using qPCR Analysis

**Figure 5:**
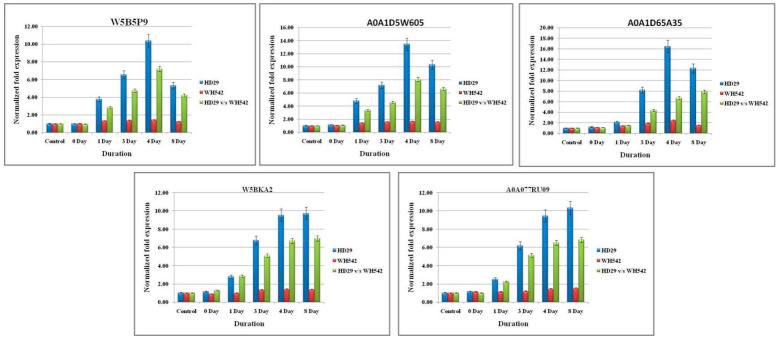
Real-time quantitative PCR (RT-qPCR) validation of RNA sequencing (RNA-Seq) data through randomly selected upregulated DEGs in the wheat infection. Relative fold change values were generated for RT-qPCR samples by comparing the expression of genes at each time point of infected resistant (HD29) vs. susceptible (WH542) wheat varieties. Data are presented as means ± standard error (SE) from three independent replicates.

**Figure 6:**
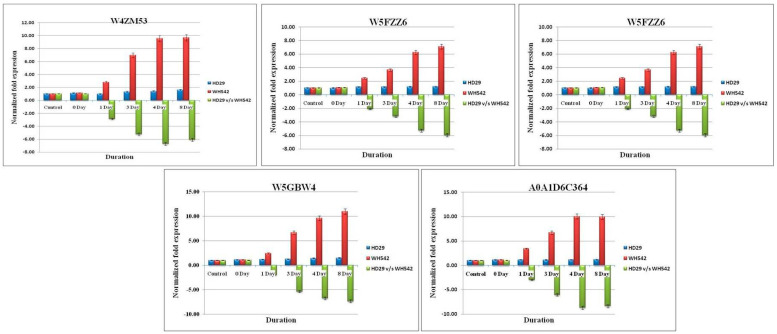
Real-time quantitative PCR (RT-qPCR) validation of RNA sequencing (RNA-Seq) data through randomly selected downregulated DEGs in the wheat infection. Relative fold change values were generated for RT-qPCR samples by comparing the expression of genes at each time point of infected resistant (HD29) vs. susceptible (WH542) wheat varieties. Data are presented as means ± standard error (SE) from three independent replicates.

A total of 10 transcripts were subjected for differential gene expression (DEGs) between the resistant (HD29) and susceptible (WH542) genotypes. Ten randomly taken DEGs (five upregulated and downregulated DEGs; Table 5) were validated using the gene-specific primers through qPCR. The expression level of these DEGs was similar to the level of expression in RNA-Seq analysis at different time points (0, 1, 3, 4, and 8 days, respectively) (Figure 5 and Figure 6).

Although a significant and similar pattern of expression was found for all the selected genes wherein the expression level was different in terms of fold changes. The expression level of five-selected upregulated genes (W5B5P9, A0A1D5W605, A0A1D65A35, W5BKA2, and A0A077RU09) were found in the range of 6.8- to 8.06-fold higher in the resistant genotype (HD29) with respect to the same time point of susceptible genotype (WH542). The highest upregulation was found in the transcript profile of A0A077RU09 and A0A1D65A35 resistant genotype with 8.06- and 7.9-fold increases, respectively, as compared to susceptible genotype as control. The pattern of upregulation was found to be similar for all selected genes wherein the highest-level expression was observed on the 4^th^ to 8^th^ days. Within the *T. indica*-inoculated wheat genotype (HD29), the highest upregulation was also observed for A0A1D65A35 and A0A077RU09 transcripts with 16.46- and 13.46-fold increase, respectively, against the control (un-inoculated HD29 genotype), while no significant upregulation was observed in the WH542 samples. On the other hand, five selected downregulated genes likewise patterns were in an opposite manner of upregulated genes. In downregulated genes viz. W4ZM53, W5A6W5, W5FZZ6, W5GBW4, and A0A1D6C364, the highest downregulation was observed in the transcripts of A0A1D6C364 and W5GBW4 in the resistant genotype) with −8.87- and −7.36-fold, respectively.

## 3. Discussion

Karnal bunt of wheat is the most serious disease incited by *T*. *indica*. The genome sequencing of *T*. *indica* revealed a molecular basis of a unique mode of infection as well as survival and the dormancy of teliospores. However, when it comes to studying the plant-pathogen interaction, in-depth transcriptomic analysis is crucial to revealing the molecular mechanisms that control disease progress [24]. Plant–pathogen interaction is a very complex process that triggers many molecular responses at different levels of infection, mainly in bunt pathogens infecting wheat. Until now, no information on wheat-*T*. *indica* interaction transcript levels has been available upon infection. Therefore, it is essential to understand transcripts in the host–pathogen interaction stress [25]. The transcriptome analysis investigated wheat crops with other bunt pathogen infection against *T*. *laevis* [26] and *T*. *controversa* [27]. Keeping this in view, the transcriptome analysis of wheat–*T*. *indica* interaction in resistant and susceptible genotypes was conducted to understand the resistance mechanism

The establishment of pathogen infection in the host plant is a prerequisite for any transcriptomic study investigating the plant–pathogen interaction. In the present study, *T. indica* was inoculated at the boot leaf stage of the wheat plant, which resulted in spikes with severe Karnal bunt symptoms after 3 days of inoculation. The spikes were found to be ideal for the transcriptomic analysis of wheat-*T*. *indica* interaction after 3 days of inoculation. Hence, two time points (0 and 1 day) before and two time points (4 and 8 days) after were taken into account for detailed investigation. In one of the previous few studies on the infection mechanism of *T*. *indica*, it was established through histo-pathological and radiotracer techniques that the pathogen favors the boot stage for infection [28]. It could also successfully establish the Karnal bunt infection after 3 days of inoculation at the boot leaf stage of wheat plants [29].

Upon the fungal plant infection, plants trigger different defense-related genes to overcome the disease severity in different crops [27]. Few defense-related genes against *T*. *indica,* such as Puroindoline protein PINB, β-1,4-glucanase, and chitinase were highly expressed in resistant host as compared to susceptible host [30]. The genes encoding chitinase, lipase, and defensins were expressed in wheat crops upon *T*. *controversa* infection [31]. Defense genes play an important role in overcoming the severity of disease. In our study, 118 defense-related genes were identified, which encode the pathogen recognition, defense response to fungus, response to biotic stimulus, and metal ion binding. Additionally, responses to stimulus (uncharacterized protein in wheat) belonging to cell surface receptor signaling pathway and stress genes (peroxidase and uncharacterized protein) were significantly expressed in resistant genotypes only. These transcriptional changes may have a role in the resistance against *T*. *indica* inciting Karnal bunt of wheat.

In the present study, the DEGs could be classified into three functional categories viz., the biological processes, molecular functions, and cellular components. In the biological process, the majority of the DEGs were related to stimulus response, defense response to fungus, catabolic process, and response to wounding. In the molecular functions, most of the DEGs were related to metal ion binding, transferase activity, DNA binding, ATPase activity, heme binding, and sequence-specific DNA binding. Further, in the cellular component category, most of the DEGs were related to the integral component of the membrane, extracellular region, carbohydrate metabolic process, and nucleus. Similar findings were obtained on the RNA-seq analysis of tomato-*Verticillium dahliae* interaction, where the DEGs of response to stimuli were highly associated with biological processes [32,33]. However, unlike in this study, the DEGs for metabolic and cellular processes were also highly associated with the biological processes instead of the cellular components. This indicates the different array of expression exhibited by similar gene types upon *T*. *indica* infection in resistant as well as susceptible genotypes of wheat, respectively. This was evident in the present study as only a few genes, such as like integral component of membrane, exonuclease activity, nucleic acid binding, DNA binding, metal ion binding, ATP binding, methionine adenosyl transferase activity, and transferase activity differed in their expression under both resistant and susceptible genotypes. 

In the functional annotations, DEGs were mainly involved in the important biological functions, such as signal transduction, secondary metabolite biosynthesis, and responses to stimulus. It is a well-established fact that signal transduction during plant-pathogen interactions largely depends on the biosynthesis of various secondary metabolites. These findings suggest that the biosynthesis of secondary metabolites upon *T*. *indica* infection at the boot stage of wheat induces signal transduction pathways leading to either expression or repression of the pathogenesis-related (PR) protein genes. Fungal growth, development, infection, and virulence are dependent on signaling cascades [34]. The biosynthesis of secondary metabolites upon *Verticillium dahliae* infection induced the signal transduction pathways leading to the expression of PR protein genes in the resistant tomato genotypes and repression in the susceptible genotypes [35]. Few pathogenicity-related genes, such as chitinase and MAP kinase *Hog1*, were characterized in *T*. *indica* [36,37].

In our present investigation, the KEGG analysis identified 208 pathways involved in 22 functional groups, mainly plant hormonal signal transduction, plant–pathogen interaction, phenyl-propanoid biosynthesis, and translation during plant-pathogen interaction. The maximum number of DEGs were categorized into amino acid metabolism, biosynthesis of secondary metabolites, and carbohydrate metabolism. Amino acids played crucial roles in plants, such as stress defense, development, and acting as hormone precursors [38,39]. During the plant–pathogen interaction, the phenyl-propanoid pathway has been reported to play a critical role in plant defense against pathogenic fungi [40]. Certain genes exclusively involved in a phenyl-propanoid pathway have also been reported to induce a compatible interaction of lettuce and *Botrytis cineraria* [41].

## 4. Materials and Methods

### 4.1. Fungus Material and Inoculum Preparation

Wheat Karnal pathogen *T*. *indica* isolate RAKB_UP_1 (NCBI *Genbank* accession-KX369242) was used for the present study. The isolate was cultured on potato dextrose agar (PDA) media manufactured by HiMedia Laboratories Pvt. Ltd., India and incubated at 16 ± 2 °C under 12 h of light/dark conditions in the incubator. The inoculum of secondary sporidia at a concentration of 1 × 10^4^ sporidia mL^−1^ was prepared after three weeks with the help of the hemocytometer under microscope.

### 4.2. Plant Growth Conditions and Inoculation

Healthy seeds of wheat genotypes viz. HD29 (resistant) and WH542 (susceptible) were taken from Division of Genetics, ICAR-IARI, New Delhi, India. Plants were grown under the net house conditions during the *rabi* season (November–April) and raised up to the boot leaf stage (Z-49 stage). Inoculation of *T. indica* was done by injecting sporidia (ca. 1 × 10^4^ sporidia mL^−1^) using a sterile syringe in the boot of wheat spikes [42,43]. After inoculation, infected boot stage samples were collected at different time points after inoculation, viz. 0 days, 1 day, 3 days, 4 days, and 8 days, and stored at −80 °C for further study.

### 4.3. RNA Extraction and Library Construction

Transcriptome analysis of two wheat genotypes viz. HD29 (resistant) and WH542 (susceptible) inoculated with *T*. *indica* at boot stage was performed. RNA sequencing (RNA-seq) of RNA isolated from infected wheat spikes was used. Total RNA was isolated from all the infected samples using the TRizol method with minor modifications. The quality of the isolated RNA was checked on a high-sensitivity RNA D1000 Screen Tape, and the quantifications were done using a Qubit 3.0 fluorometer, Thermo Fisher, US. RNA samples from each group (resistant and susceptible at different time points) were pooled in an equimolar concentration for library preparation. The RNA-Seq paired-end sequencing libraries were prepared from the total RNA using Illumina TruSeq stranded mRNA sample preparation kit, Illumina Inc, US. Briefly, mRNA was enriched from the total RNA using poly-T attached magnetic beads, followed by the enzymatic fragmentation and 1st strand cDNA conversion. The 1st strand cDNA was then synthesized to the second strand using the second strand mix and Act-D mix to facilitate RNA-dependent synthesis. The ds-cDNA samples were then purified using Ampure XP beads followed by A-tailing, adapter ligation, and then enriched by the limited number of PCR cycles.

### 4.4. Illumina Sequencing and Reads Mapping

The sequenced raw data was processed to obtain the high-quality clean reads through the Trimmomatic v0.35 (http://www.usadellab.org/cms/?page=trimmomatic, accessed on 1 June 2021) to eliminate adapter sequences, ambiguous reads, and low-quality sequences [44]. A minimum length of 100 nucleotides (nt) after trimming was applied. High-quality reads obtained from both the resistant and susceptible genotypes were applied for mapping with a reference genome of *Triticum aestivum* (Taxon ID: 4565) using BWA mem, version: 0.7.12-r1039 (https://github.com/lh3/bwa, accessed on 1 June 2021) with default parameters.

### 4.5. Analysis of Differentially Expressed Genes (DEGs)

The differential gene expression analysis was performed using the samtools (v 0.1.18) and bedtools (v 2.17.0) (http://www.htslib.org/, accessed on 1 June 2021) to count the number of reads mapped on individual genes. The gene name and coordinate information were obtained from the Ensembl GFF (gene feature file) file of *T. aestivum*. A gene was defined as expressed if more than five reads were mapped. The genes having less than an arbitrary threshold of five mapped reads were filtered out.

A Benjamin-Hochberg correction for multiple testing was performed using DESeq to identify the significantly expressed genes. These genes were further divided based on their statistical significance (which can be either “yes” or “no”), depending on whether the *p*-value is less than 0.05 and the false discovery rate (FDR) is 0.05. The FDR corrected significant gene expression analysis was carried out considering the Log_2_ fold values (Log_2_ fold value > 0 for upregulation and Log_2_ fold value < 0 for downregulation) and significance “yes” for *p*-value less than 0.05.

The hierarchical cluster analysis was conducted on the top 100 DEGs through a multiple experiments viewer, MeV v4.9.0 (https://mev.tm4.org/#/about, accessed on 1 June 2021). A heat map was generated using the log-transformed and normalized values of genes based on the Euclidean distance as well as the average linkage method. The expression levels were represented as log_2_ ratios of gene abundance between the resistant and susceptible genotypes. The Eurofins proprietary R script was used for the graphical representation of DEGs, distributed among the resistant and susceptible genotypes in a volcano plot.

### 4.6. Functional Annotation and Gene Ontology Enrichment Analysis

The functional annotations of genes were carried out against the curated KEGG GENES database using the KAAS, KEGG Automatic Annotation Server (https://www.genome.jp/kegg/kaas/, accessed on 1 June 2021) [45]. The KEGG Orthology (KO) was assigned to each gene to associate with its KEGG metabolic pathway. The KEGG orthology database of *Arabidopsis thaliana* was taken as a reference for the pathway mapping using the bidirectional best hit (BBH) method.

A further study, the gene ontology (GO) enrichment analysis, was done using the singular enrichment analysis (SEA) of agriGO (http://bioinfo.cau.edu.cn/agriGO; accessed on 1 June 2017)) to target three main domains, namely cellular component, molecular function, and biological process. The GO term enrichment of target genes was computed by comparing them with *T*. *aestivum* reference genes using the SEA analysis. The hypergeometric tests with the Hochberg FDRs were performed using the default parameters to adjust the *p*-value < 0.05 for obtaining the significant GO terms.

### 4.7. Differential Expression Analysis in T. indica upon Infection

The high-quality reads of both the resistant and susceptible genotypes were mapped against *T. indica* genome RAKB_UP_1 (MBSW01000000) using the BWA mem (Version: 0.7.12-r1039) aligner. The differential gene expression analysis of *T. indica* genes was carried out using samtools v 0.1.18 (http://www.htslib.org/, accessed on 1 June 2021) between the susceptible and resistant genotypes to count the number of reads mapped on individual genes. A gene was defined as expressed if more than five reads were mapped. Those genes that had less than an arbitrary threshold of five mapped reads were filtered out.

### 4.8. Validation of Differentially Expressed Genes by Quantitative Real-Time PCR

The expression profile of ten randomly selected DEGs at different time points was analyzed using a two-step real-time qPCR. The primers were designed using the Integrated DNA Technologies (IDT) primer designing tool. The designed primers were synthesized from GCC Biotech Pvt., West Bengal, India (Table 5). Total RNA and cDNA synthesis were performed as described in a previous study [46]. The qPCR was carried out in a real-time PCR system (BioRad). The qPCR conditions were standardized to identify the ideal Tm of primers for each selected gene in a gradient thermal cycler. The SYBR green was used as a detection dye. The SYBR Green PCR Master Mix (Thermo Fisher, US) was used. The reaction was set up in a final volume of 20 μL. The standard thermal profile was used: pre-incubation (95 °C for 15 min), denaturation (95 °C for 15 s), annealing, and extension (60 °C for 1 min with 40 cycles of amplification). The *RPS17* gene was used as a reference for normalization. The gene expression was normalized by subtracting the mean C_T_ values for a reference gene *RPS17* from ΔC_T_ values of DEGs. The fold change values were calculated (ΔΔC_T_ represent = ΔC_T_ condition of target gene—ΔC_T_ control gene) according to the 2^−ΔΔCT^ method [47].

## 5. Conclusions

Transcriptome analysis suggested that most of the DEGs involved in the responses to stimuli and metabolic processes were expressed upon *T*. *indica* infection. In the molecular functions, DEGs were the binding and catalytic activity. The KEGG pathway analyses revealed the DEGs involved in the signal transduction, carbohydrate metabolism, and energy metabolisms affected during early infection of the disease. The present results may help in finding new markers to develop resistant varieties. It also provides interesting information on gene expression patterns in wheat, upon *T*. *indica* infection, revealing the molecular signaling mechanisms and complex regulatory processes. The functional characterization of the DEGs identified will further help researchers to understand the resistance and infection mechanism of *T*. *indica*. This is the first report on global transcriptome analysis of wheat-*T*. *indica* interaction, which will further provide insights in host-pathogen interaction.

## Figures and Tables

**Table 1:** Summary statistics of reads generated in RNA-Seq analysis of resistant and susceptible genotypes of wheat upon *T*. *indica* infection.

Description	Resistant Genotype (HD29)	Susceptible Genotype (WH542)
Total Reads	29,321,161 (100%)	29,062,661 (100%)
Number of bases	8,611,180,199	8,531,647,691
Total data in Gb	8.6 Gb	8.5 Gb
Mapped reads	93.67%	92.11%
Uniquely mapped reads	92.94%	90.47%
Multiple mapped reads	0.72%	1.63%
Unmapped reads	6.32%	7.88%
% GC content	51.38%	49.48%

**Table 2:** Gene ontology descriptions of significantly upregulated genes.

GO Terms	Ontology	Descriptions	Total Input List
GO:0050896	P	response to stimulus	13
GO:0006950	P	response to stress	11
GO:0005975	P	carbohydrate metabolic process	9
GO:0008152	P	metabolic process	21
GO:0042221	P	response to chemical stimulus	7
GO:0009628	P	response to abiotic stimulus	5
GO:0044238	P	primary metabolic process	17
GO:0032502	P	developmental process	5
GO:0009056	P	catabolic process	6
GO:0065007	P	biological regulation	7
GO:0004553	F	hydrolase activity, hydrolyzing O-glycosyl compounds	7
GO:0016798	F	hydrolase activity, acting on glycosyl bonds	7
GO:0005488	F	Binding	23
GO:0003824	F	catalytic activity	19
GO:0016787	F	hydrolase activity	9

**Table 3:** Mapping statistics with *T. indica* genome.

Particulars	Number of Mapped Reads	% Mapping
Resistant genotype (HD29)	4,146,257	7.07
Susceptible genotype (WH542)	4,434,294	7.63

**Table 4:** DESeq analysis of *T. indica* up on infection.

Descriptions	Number of Genes
Exclusive susceptible (HD29) genotype	1271
Exclusive resistant (WH542) genotype	326
Gene expressed both resistant and susceptible hst (HD29 & WH542)	7704
Upregulated	221
Downregulated	146
FDR-Upregulated	11
FDR-Downregulated	09

**Table 5:** List of differentially expressed genes used for expression analysis.

Gene IDS	Log2 Fold Change	UniProtKB/TrEMBL ID	GO Terms	Forward Primer (5′-3′)	Reverse Primer (5′-3′)	Protein Name	Functions
**Five Up Regulated Genes in Wheat-*T. indica* Interaction**							
TRIAE_CS42_2AS_TGACv1_115171_AA0371320	6.590434	W5B5P9	Integral component of membrane	GTGATGTGTCCATTGCATGTG	AAACGCAGTCCATATAGCCAG	Uncharacterized protein	-
TRIAE_CS42_3B_TGACv1_224664_AA0799640	9.915167	A0A1D5W605	-	CGCAGCTTATCACGACAATG	GCTAGTAAAAGGGACAGGGAC	Uncharacterized protein	-
TRIAE_CS42_6BS_TGACv1_513159_AA1632520	8.262696	A0A1D65A35	3′-5′ exonuclease activity, nucleic acid binding	GAATCCACCTCTCACCATCC	ACGGAACAGACCAGCATAAG	Uncharacterized protein	-
TRIAE_CS42_2BS_TGACv1_147826_AA0488000	6.692584	W5BKA2	integral component of membrane	ACGGCCTCAGAGCTTATGTG	GGTTTGCACTTGACCACAACA	Uncharacterized protein	-
TRIAE_CS42_3B_TGACv1_221012_AA0726950	6.968122	A0A077RU09	DNA binding, protein heterodimerization activity	TCACCTCCTCGTAGAGTGCT	AGGCAGGCACCGTTACTATT	Histone H2B	Core component of nucleosome. DNA repair, DNA replication and chromosomal stability.
**Five Down Regulated Genes in Wheat-*T. indica* Interaction**							
TRIAE_CS42_1AL_TGACv1_000260_AA0007580	−7.39315	W4ZM53	transferase activity, transferring acyl groups other than amino-acyl groups	GCGGCTTTCAATCTTTTCAGG	TGCTCTGTTTGGTGATGGTG	Uncharacterized protein	-
TRIAE_CS42_1BL_TGACv1_030856_AA0102410	−5.23889	W5A6W5	serine-type, carboxypeptidase activity	AACTTGCTGTTCCTGGACTC	TCGCTAACTGTGGAATGTAGTG	Carboxypeptidase	cleave peptides from the C-terminus at a speed of one residue each time
TRIAE_CS42_5DL_TGACv1_435993_AA1457050	−5.12949	W5FZZ6	coenzyme binding	TCTTCCACATTGCATCACCTG	GTTTCGTCCCTAATCCTCACTG	Uncharacterized protein	-
TRIAE_CS42_6BS_TGACv1_515607_AA1671270	−7.7181	W5GBW4	metal ion binding, ATP binding, methionine, adenosyl transferase activity	CACAATGACAATGGTGCTATGG	ATGGGTTGAGATGGAAGATGG	S-adenosylmethionine synthase (EC 2.5.1.6)	Catalyzes the formation of S-adenosylmethionine from methionine and ATP.
TRIAE_CS42_7BL_TGACv1_576986_AA1861990	−9.32268	A0A1D6C364	-	CTTGTCTTATTCCCTCACCCAC	ACCTGACCCCTAATTGCTTG	Uncharacterized protein	-

## Data Availability

The data presented in this study are available on the NCBI SRA database (SRP159223).

## Supplementary Materials

The following supporting information can be downloaded at: https://www.mdpi.com/article/10.3390/plants11223061/s1, Table S1: Gene expression analysis with annotation in the resistant (HD29) and the susceptible (WH542) genotypes; Table S2: Significantly expressed GO terms in both the genotypes; Table S3: Significantly expressed GO terms in the resistant genotype (HD29); Table S4: Significantly expressed GO terms in the susceptible genotype (WH542); Table S5: Identified KEGG pathways and functional categories; Table S6: Differentially expressed defense-related genes; Table S7: Gene expression analysis with annotation with *T. indica* in the resistant and susceptible genotypes.
